# Computer-aided molecular and biological-immune modeling of *illicium verum* bioactive compounds employing the Egyptian Nile snail* Biomphalaria alexandrina* as a paradigm

**DOI:** 10.1007/s10822-025-00607-2

**Published:** 2025-06-20

**Authors:** Alya Mashaal, Basma M. Abou El-Nour, Fatma M. Ismail, Eman A. Elewa, Eman A. Elnoby, Eman B. Ebada, Ayaat G. Mohammed, Manar F. El-Sahmawy, Mariam M. Mansour, Nermeen N. Khames, Hend M. Ghorab, Safaa A. Osman, Alshimaa A. Elsaid, Maryam M. Abd-Alaziz, Asmaa S. Zayed, Asmaa A. Abo Elqasem

**Affiliations:** 1 Immunology, Zoology and Entomology Department, Faculty of Science, Al- Azhar University (Girl’s Branch), Cairo, Egypt; 2 Parasitology, Zoology and Entomology Department, Faculty of Science, Al- Azhar University (Girl’s Branch), Cairo, Egypt; 3 Special Zoology, Zoology and Entomology Department, Faculty of Science, Al- Azhar University (Girl’s Branch), Cairo, Egypt

**Keywords:** *Illicium verum* (star anise), Biological activities, *Biomphalaria alexandrina* model, Oxidative–immune mediators, Gene‒protein interaction, Computer-aided molecular simulation

## Abstract

In pursuit of sustainable biocontrol strategies, this study explores *Illicium verum* (star anise) as a dual-action anti-inflammatory/oxidative and molluscicidal agent using *Biomphalaria alexandrina,* the intermediate host of *Schistosoma mansoni,* as an eco-relevant in vivo model. Two experimental snail groups were employed: a control group and a treatment group exposed to a sublethal concentration of *I. verum* extract (LC₁₀ = 315 ppm). Through a combined pipeline of phytochemical profiling, computational simulations, and in vivo assays, we identified flavonoids and phenylpropanoids with potent bioactivity. Molecular docking and ADMET screening highlighted kaempferol, quercetin, and rutin as top ligands, which bind effectively to key snail proteins such as cytochrome c oxidase and actin. In vivo analyses confirmed immunomodulatory effects, and these findings were validated through oxidative/inflammatory biomarker assays, which revealed altered cytokine levels (IFN-γ, IL-2 and IL-6), tissue remodeling, and reduced oxidative stress. Histopathological and immunohistochemical evaluations revealed significant tissue alterations in the digestive gland and head-foot regions of treated snails. Gene and protein interaction networks supported these findings by linking compound action to immune and oxidative regulatory pathways. This integrative study demonstrated that *Illicium verum* contains bioactive compounds capable of modulating oxidative stress, immune responses, and tissue integrity in *B. alexandrina* as an animal model. Integrating phytochemical analysis with in silico and molecular simulations offers a powerful approach for understanding and optimizing bioactive compounds. While phytochemical profiling identifies key constituents such as flavonoids and phenylpropanoids, computational tools predict their binding to biological targets, pharmacokinetics, and safety. This combination not only streamlines the discovery of effective and low-toxicity compounds but also clarifies their mechanisms of action at the molecular level, enhancing both the precision and efficiency of experimental validation. These results position star anise as a promising, eco-friendly candidate for the development of novel molluscicidal and anti-inflammatory agents supporting sustainable disease control strategies.

**Graphical Abstract:**

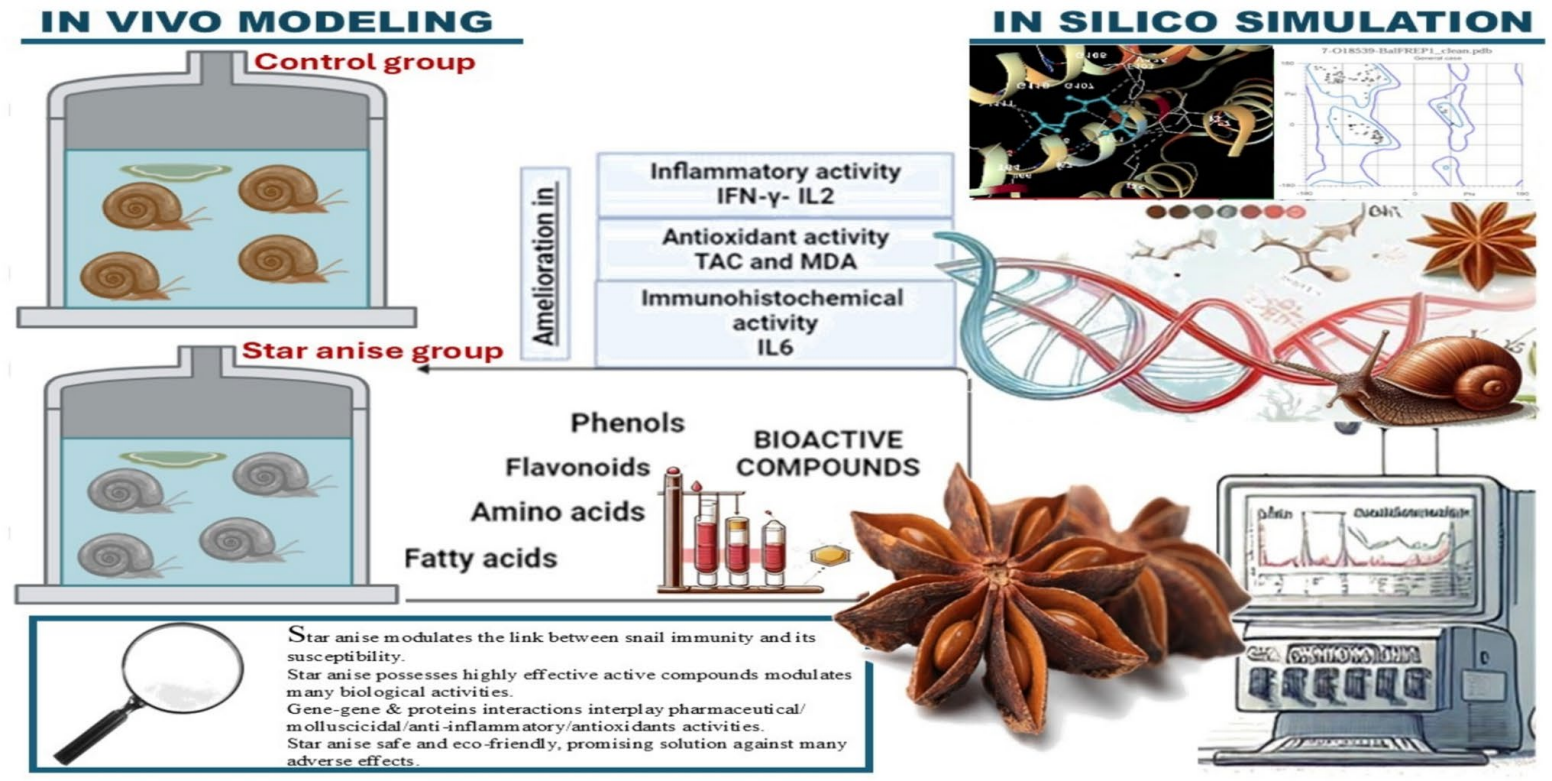

**Supplementary Information:**

The online version contains supplementary material available at 10.1007/s10822-025-00607-2.

## Introduction

Medicinal plants are promising alternatives for treating various diseases and serve as rich reservoirs of bioactive compounds with potent pharmacological effects and minimal side effects [1]. Among these, many exhibit immunomodulatory properties, providing sustainable and natural means for immune system modulation [2]. Their role is increasingly recognized in contemporary healthcare, particularly in phytotherapy and sustainable aquaculture, where natural agents such as essential oils (EOs) rich in terpenes, terpenoids, phenylpropenes, and isothiocyanates, exhibit strong biocidal effects against pathogens [3].

*Illicium verum* (star anise) has garnered attention for its wide array of pharmacologically active constituents, including those with significant immunomodulatory potential [4]. In traditional natural medicine, star anise has promising therapeutic applications through its antioxidant, antimicrobial, anti-inflammatory, antifungal, antinociceptive, anthelmintic, insecticidal, secretolytic, gastroprotective, sedative, expectorant, spasmolytic, and estrogenic effects [5, 6]. Anethole, its major component (~ 90%), along with estragole and anisaldehyde, contributes to these bioactivities while retaining the characteristic aroma of the plant [7, 8].

Despite these benefits, concerns about pesticide toxicity have limited broader applications in pest management. However, recent investigations have shown its potential in modulating immune responses during parasitic infections, such as its effect on cytokine release in *Lernaea cyprinacea* infections [9]. With increasing concern over the ecological risks posed by chemical molluscicides such as threats to water quality and aquatic life natural remedies such as star anise are increasingly favored [10]. Historically, herbal remedies have also played a vital role in treating parasitic diseases, especially in regions with limited access to conventional healthcare [11].

*Biomphalaria alexandrina* is widely recognized as a valuable invertebrate model because of its unique biological features, adaptability, and relevance to multiple scientific domains [12]. These snails have supported research in neurobiology, ecology, pharmacology, developmental biology, physiology, genetics, and evolutionary fields [1]. The high reproductive capacity and resilience of these plants in various environments further strengthen their role in laboratory experimentation [13].

Gastropods, including *B. alexandrina*, possess a specialized internal defense system (IDS) distinct from vertebrate immunity. This system comprises cellular and humoral components, such as hemocyte phagocytic cells found in hemolymph, which mediate immune responses through encapsulation, phagocytosis, and secretion of cytotoxic molecules [12]. Hemocytes also engage in oxidative burst reactions that generate reactive oxygen species (ROS) via NADH oxidase activity [14]. Additionally, they release cytokines and immune effectors such as agglutinins and antimicrobial peptides to regulate immunity [15].

Given their sensitivity to environmental changes, snails like *B. alexandrina* are excellent bioindicators for ecological studies. Its use in assessing the toxicological effects of environmental contaminants is well documented [1, 13]**.** Biomphalaria’s accessibility, ease of maintenance, and responsiveness to water pollutants make it ideal for ecotoxicological monitoring [16]. Its utility as a model organism has been affirmed across disciplines, including immunology, reproductive, and developmental biology [12]**.** In a pioneering effort, this study incorporates computational simulation techniques to evaluate the interaction of Illicium verum phytoconstituents with key protein targets of *B. alexandrina*, providing mechanistic insights into their bioactivity. This work uniquely bridges traditional plant-based knowledge with molecular simulations and protein‒target interaction analyses. This study further advances the field by targeting specific proteins involved in snail physiology and the immune response, offering mechanistic insights and identifying promising compounds that could serve as eco-friendly alternatives in schistosomiasis control strategies.

## Materials and methods

### Herbal material

Dried fruits of *Illicium verum* were obtained from Imtenan Healthy Company, Cairo, Egypt, and described as being free from pesticides, microbial contamination, and plague infection. These conditions ensure that the botanical material is highly pure and safe for both phytochemical and biological evaluation, minimizing the risk of interference from external contaminants in the experimental outcomes. The fruits were finely ground into a powder via an electric mill to facilitate efficient extraction. For the extraction process, the powdered material was suspended in warm distilled water and subjected to continuous extraction under controlled conditions at 37 °C for 18 h under slight pressure, as previously described by Sabry et al. [17]. After extraction, the aqueous solution was carefully filtered through a sterile 0.22 μm membrane filter to ensure sterility, and any particulate matter was removed. The sterile filtrate was then lyophilized using a Dura-Dry MP freeze-dryer (FTS Systems, USA) to obtain a dry, stable powder form of the extract. The resulting lyophilized extract was stored at 4 °C in sterile airtight containers until used for subsequent phytochemical analyses.

### Assessment of total phenolic and flavonoid contents

The total phenolic content was determined using a modified folin-ciocalteu assay, the method described by Lawag et al. [18]. Briefly, 0.1 g of lyophilized powder was dissolved in 1 ml of deionized water. Then, 1 ml of the extract was mixed with 2.5 ml of deionized water, followed by the addition of 2.5 ml of Folin–Ciocalteu reagent (10%, v/v). After the mixture was incubated for 5 min at room temperature, 2 ml of sodium carbonate solution (7%, w/v) and 2 ml of deionized water were added. The mixture was then thoroughly vortexed and incubated in the dark at 25 °C. The absorbance was measured at 760 nm via a UV/Vis spectrophotometer (Hitachi U-2900). Gallic acid served as the standard, with a calibration curve prepared in the range of 0–40 ppm. The TPC was expressed as milligrams of gallic acid equivalents per 100 mg of extract (mg GAE/100 mg).

A colorimetric approach utilizing aluminum chloride was employed to determine the total flavonoid content of the extracts, following the method described by Beyhan & İşleroğlu [19] with some modifications. Initially, a mixture of 1 ml of extract, 4.1 ml of distilled water, and 0.3 ml of 5% NaNO2 was incubated for 5 min. Subsequently, 0.1 ml of 10% AlCl3 was added, followed by further incubation for 6 min. Next, 0.1 ml of 1 M NaOH solution and 2.8 ml of deionized water were added, and the mixture was incubated for 30 min in darkness. The absorbance of the sample was measured at a wavelength of 415 nm, using quercetin as the standard flavonoid and the results were expressed as mg L^−1^.

### GC‒MS analysis of Illicium verum

Gas chromatography–mass spectrometry (GC‒MS) analysis was performed using Agilent 6890 N gas chromatography system equipped with an autosampler and coupled to a mass spectrometer, was utilized. A one microliter sample was injected in pulsed splitless mode onto a fused silica column with a film thickness of 0.15 µm and dimensions of 30 m × 0.25 mm ID. Helium gas served as the carrier gas, maintaining a constant flow rate of 1 ml/min, while the column head pressure was maintained at 20 psi. The column temperature profile began at 55 °C for 0.4 min, followed by an increase to 200 °C at a rate of 25 °C per minute, then to 280 °C at a rate of 8 °C per minute, and finally to 300 °C at a rate of 25 °C per minute, where it was held for 2 min. Compound identification was achieved by comparing the obtained data with authentic reference standards and published data according to the data described by **Sabry et al. **[17]. The relative abundance of each compound was calculated on the basis of its corresponding GC peak area.

### Computational methodology

#### Biological activity of Illicium verum chemical compounds

Biological activities were determined via compound nomenclature obtained from the GC‒MS results, employing the Open Parser for Systematic IUPAC nomenclature (OPSIN^©^), Centre for Molecular Informatics, University of Cambridge, UK; then, the computed representation of chemical structure was employed using a chemical notation SMILES. A simplified Molecular Input Line Entry System, which is used to draw chemical structures, was used, employing Avogadro (1.2.0n) © 2022 Avogadro Chemistry according to the methodology of **Saha et al. **[20]. Finally, prediction of biological activities was performed via Pass online (Way2Drug^©^, Version 2.0) according to the methodology of **Ferdous et al. **[21], Bioinformatics Department, Structure‒Function-Based Drug Design Laboratory, Institute of Biomedical Chemistry (IBMC), Russia.

#### In silico* evaluation of ADME properties and toxicity*

The pharmacokinetic behavior and cytotoxicity of the selected active compounds were evaluated according to the method of **Alamri et al. **[22] using the SwissADME open-access web tool (http://www.swissadme.ch/) based on their highest biological probability. This platform provides predictions for key ADME parameters, absorption, distribution, metabolism, and excretion as well as additional properties such as physicochemical characteristics, lipophilicity, water solubility, pharmacokinetics, drug-likeness, and medicinal chemistry relevance, all of which are documented in the **supplementary file**.

#### Screening criteria

To identify the active compounds (ligands), an initial pool of 15 candidates was selected for the study according to their high biological probability. These compounds were evaluated for their binding efficacy to target proteins, and the compounds demonstrating the strongest binding affinity, high bioavailability, and minimal toxicity were considered active candidates.

#### Illicium verum tentative molecule preparation

Tentative molecules (Ligands) were modeled using their nomenclature obtained from the GC‒MS results and based on the data outlined by **Sabry et al. **[17]**,** employing ChemDraw® Ultra version 12.0.2.1076 software according to **Mills **[23]⁠. Subsequently, Avogadro version 1.2.0n software was utilized to optimize the ligands to a stable conformation. The optimized ligands were processed in PDB format, which was confirmed from the AlphaFold Protein Structure Database, and further analysis and use in molecular docking simulations were performed.

#### Protein (macromolecule) preparation

The specific selection of *B. alexandrina* proteins is essential because this species serves as the direct in vivo host model in the current study. Investigating its protein targets provides mechanistic insight into how bioactive compounds interact at the molecular level, supporting the study’s goal of developing effective, host-targeted molluscicidal and immunomodulatory agents.

To investigate the molecular interactions of *B. alexandrina* proteins with selected ligands, a comprehensive protein preparation pipeline was established. Relevant protein-coding genes were initially identified through the NCBI (https://www.ncbi.nlm.nih.gov/) and UniProt (https://www.uniprot.org/) databases by querying species-specific entries and reviewing annotated protein functions.

Three-dimensional structures of the selected proteins were retrieved from the AlphaFold protein structure database (https://alphafold.ebi.ac.uk/). These predicted structures were validated and prepared in PDB format for docking, ensuring the removal of water molecules and the addition of polar hydrogen atoms.

#### Protein validation

The selected proteins were validated through multiple steps: protein sequences were retrieved from the NCBI and UniProt databases and then cross-checked for accuracy and biological relevance. The corresponding 3D structures were obtained from the AlphaFold protein structure database. Functional validation was performed using QuickGO annotations to confirm each protein’s molecular function, cellular component, and involvement in relevant biological pathways, ensuring their suitability for downstream molecular docking analyses.

The Ramachandran plot was generated according to the method of **Das et al. **[24] to assess the stereochemical quality of the protein structure by analyzing the distribution of backbone dihedral angles phi (φ) and psi (ψ). The uploaded PDB file was submitted to the MolProbity web server (http://molprobity.biochem.duke.edu/index.php), where standard structure validation tools were applied. The resulting Ramachandran plot visualized the allowed and disallowed conformational regions, providing insight into the overall backbone geometry and identifying any outlier residues that may indicate structural anomalies or errors.

The functional annotation of each protein, including its molecular function and cellular component, was documented using the QuickGO database (https://www.ebi.ac.uk/QuickGO/term/), tracing associated biological pathways and ontological classifications. This comprehensive approach ensured the structural and functional integrity of protein models used in the docking studies.

### Molecular docking

The interactions of six selected ligands (phytocompounds, as described in the earlier section) with *B. alexandrina* proteins were investigated according to the method of **Das et al. **[24] via molecular docking simulations using the AutoDock Vina 1.2.0 [25]⁠. This ensured that the selected compounds were evaluated at the receptor’s active site. The docked scores were recorded, and this methodology facilitated an accurate assessment of the compounds’ binding affinities and identification of potential lead compounds for further development. CB-Dock2 was employed to visualize the molecular docking, integrating cavity detection (Å3) and predicting binding sites and affinities (kcal/mol) for ligand‒receptor interactions.

### In vivo* application*

*Biomphalaria alexandrina* snails were utilized as the in vivo model in this study to evaluate the molluscicidal, anti-inflammatory, and antioxidant activities of *Illicium verum* extract. Adult snails measuring 9–11 mm in diameter and weighing approximately 0.26 g were obtained from the Theodor Bilharz Research Institute (Giza, Egypt). All experimental procedures were conducted in accordance with institutional guidelines for the humane handling of invertebrates, ensuring minimal stress and ethical treatment throughout the study. In addition, the procedures adhered to standardized protocols for invertebrate experimentation, including controlled environmental conditions and proper disposal methods, reflecting a commitment to responsible and ethical scientific practice. Upon acquisition, snails were acclimated and maintained in transparent plastic aquaria, each stocked at a density of 10 snails per liter. The aquaria were filled with dechlorinated and aerated tap water and maintained under controlled laboratory conditions with a stable pH of 7.0 ± 0.2 and a temperature range of 25 ± 3  °C.

A 12-h light:12-h dark photoperiod was applied to simulate natural environmental conditions. Snails were fed ad libitum with dried lettuce leaves (0.5–1 g/10 snails), which were replenished regularly to maintain nutritional consistency. The water in the aquaria was replaced every two days to prevent the accumulation of waste and maintain optimal water quality. Dead or inactive snails were promptly removed, whereas only active, healthy individuals were selected and retained for subsequent experimental procedures. All handling and housing procedures followed the protocol adapted from **Ibrahim et al. **[26], ensuring optimal health and minimizing stress prior to bioassays.

### Experimental groups

Two groups of snails were employed in the study: the control group, which included normal nonexposed snails, and the exposed group, which was composed of snails subjected to an aqueous extract of star anise. Ten snails were cultured in triplicate (30 snails/experimental group). These snails were exposed to the star anise for 24 h, followed by a 24-h recovery period in dechlorinated tap water. Additionally, three control groups of the same size were immersed in dechlorinated water only, in accordance with the protocol outlined by **Ibrahim et al. **[26]. Deceased snails were removed daily, and the surviving snails were assessed after 3 and 7 days.

This study utilized two main groups of *B. alexandrina* snails to evaluate the biological effects of *Illicium verum* extract: (1) a control group consisting of healthy, unexposed snails maintained under standard conditions and (2) an exposed group comprising snails treated with an aqueous extract of star anise. Each experimental group consisted of 30 adult snails divided into three replicates of 10 snails/aquarium.

For the treatment group, snails were exposed to the aqueous extract for 24 h, after which they were transferred to fresh dechlorinated tap water for a 24-h recovery phase to allow physiological normalization. The control group, which was maintained in parallel, was immersed in dechlorinated tap water only under identical environmental and feeding conditions, as described by **Ibrahim et al. **[26]**.** Throughout the experimental period, all tanks were closely monitored. Dead snails were promptly removed daily to prevent contamination or stress to the surviving individuals. Assessments were carried out on the surviving snails at two postexposure time points, 3 and 7 days, to evaluate both immediate and delayed biological responses to the treatment.

### Hemolymph collection

Hemolymph was extracted following the treatment of *B. alexandrina* snails with a sublethal concentration of *Illicium verum* LC10 at 315 ppm for 24 h, as outlined by **Ibrahim et al. **[27]. Hemolymph was collected by gently drying the shell and carefully puncturing the cephalopedal sinus via a sterile fine needle. The exuded hemolymph was collected into prechilled Eppendorf tubes and immediately kept on ice. To prevent cell aggregation and melanization, a few microliters of anticoagulant solution (e.g. citrate buffer) were added. The samples were then centrifuged at 500 × g for 10 min at 4  °C to separate the hemocytes from the plasma, and the samples were further used for the determination of inflammatory cytokines and oxidative mediators after 3 and 7 days of treatment.

### Assessment of hemocyte alterations

Following hemolymph collection from both the star anise-exposed and control groups of snails, monolayers of hemocytes (10 μl of hemolymph) were placed on clean glass slides and allowed to adhere for 15 min at room temperature. The adhered hemocytes were then fixed with absolute methanol for 5 min, followed by staining with 10% Giemsa solution (Aldrich) for 20 min, as described by **Ibrahim et al. **[1]. The slides were subsequently examined under a microscope to assess morphological changes.

### Tissue preparation

The tissues from 10 snails in each group after 3 and 7 days of treatment were weighed and then homogenized in ice-cold, double-distilled water using a glass Dounce homogenizer at a ratio of 1 g of tissue per 10 ml of water. The homogenates obtained were centrifuged at 3000 rpm for 10 min, after which the supernatants were collected and stored at -80 °C until further analysis, as outlined by Ibrahim et al. [26]. Then, the samples were applied according to the pamphlet of each oxidative mediator kit.

### PICKLE (protein‒protein interaction base)

PICKLE (Protein InteraCtion KnowLedgebasE) [28], University of Patras, Greece. Release 3.3, 1 October 2021. http://www.pickle.gr/ (Accessed on 2 March 2024) was used to predict inflammatory/oxidative interactions. The interaction is applied to INF-γ, IL-6 cytokines, that modulates the inflammatory pathway, and reactive oxygen species modulator (ROMO1) is an oxidative mediator of oxidative biomarkers. PICKLE serves as a meta-database designed for the direct simulation of the protein‒protein interactome, seamlessly integrating various publicly accessible protein‒protein interaction (PPI) databases through genetic information ontology, visualized via Cytoscape.js 3.3.0.

### Gene–gene interactions and pathway-computational simulations

To support and validate the relevance of the in vivo findings, bioinformatics analysis was performed to predict the top 15 interacting genes associated with key cytokines: IFN-γ, IL-2, and IL-6. This was conducted using the University of California Santa Cruz (UCSC) Genome Browser (RRID:SCR_005780), an established tool for exploring gene function and interaction networks. The genome browser was accessed through the Genomics Institute website [http://genome.ucsc.edu/index.html] on 18 May 2024.

Using the browser’s integrated gene interaction tools and reference annotations, cytokine-specific gene maps were generated. Interaction networks were filtered based on known co-expression, co-localization, physical interaction, and shared pathway involvement using supporting databases. The top 15 genes for each cytokine were selected on the basis of the highest interaction confidence scores. These predicted gene sets serve as a molecular basis for linking the immunomodulatory effects observed in *B. alexandrina* to conserved vertebrate cytokine signaling pathways, as previously described by **Fernandes et al. **[29].

### Inflammatory and oxidative assays

Interferon‐gamma (IFN‐γ; Cat. No. 430801) and interleukin-2 (IL-2; Cat. No. 431001) levels in the hemolymph were measured using enzyme-linked immunosorbent assay (ELISA) kits obtained from BioLegend, Inc. (ISO 13485 certified; BioLegend Way, San Diego, CA 92121). The absorbance was recorded at 450 nm via a UVmax ELISA reader (Molecular Devices Corp).

The quantification of the antioxidant biomarkers (TAC and MDA; Biodiagnostic kits, Biodiagnostic) was employed to quantify the tissue total antioxidant capacity (TAC; Cat. No. TA 2513), using assays described by **Koracevic et al. **[30] and malondialdehyde; lipid peroxide was determined (BioVision Incorporated, Catalog # K739-100) using assays described by **Ohkawa et al. **[31].

### Immunohistochemical assay

To evaluate cellular inflammatory signaling, the positive expression of interleukin (IL)-6 in the head-foot and digestive gland tissue was detected after 7 days of treatment. IL-6 Polyclonal Antibody (Anti-IL6, CAT # PA5-144,595; Invitrogen, Thermo Fisher Scientific) was used, and the related gene has been implicated in a wide variety of inflammation-associated states. The percentage of positively estimated areas of expression was determined via the image analysis software IPWIN 32.

### Histological assay

Histological investigations of snail head foot and digestive gland tissue samples were performed after 7 days of treatment by crushing the snail between glass slides, removing shell fragments, and fixing the soft tissues in alcoholic Bouin’s fluid. After dehydration and clearing, the samples were embedded in molten paraplast at 60 °C, sliced into 5 μm sections, and stained with hematoxylin and eosin [32]. The samples were subsequently mounted and examined using an Olympus system microscope equipped with an automatic camera.

### Statistical analysis

The SPSS application was used to analyze and interpret the data (Statistical Analysis for Social Science, Version 23). The significance of mean differences between groups was determined using the student’s *t*-test. Results were deemed to be statistically significant at a *p* value ≤ 0.05 and are presented as mean ± standard deviation.

## Results

### Phytochemical and active compounds

**Fig. 1:**
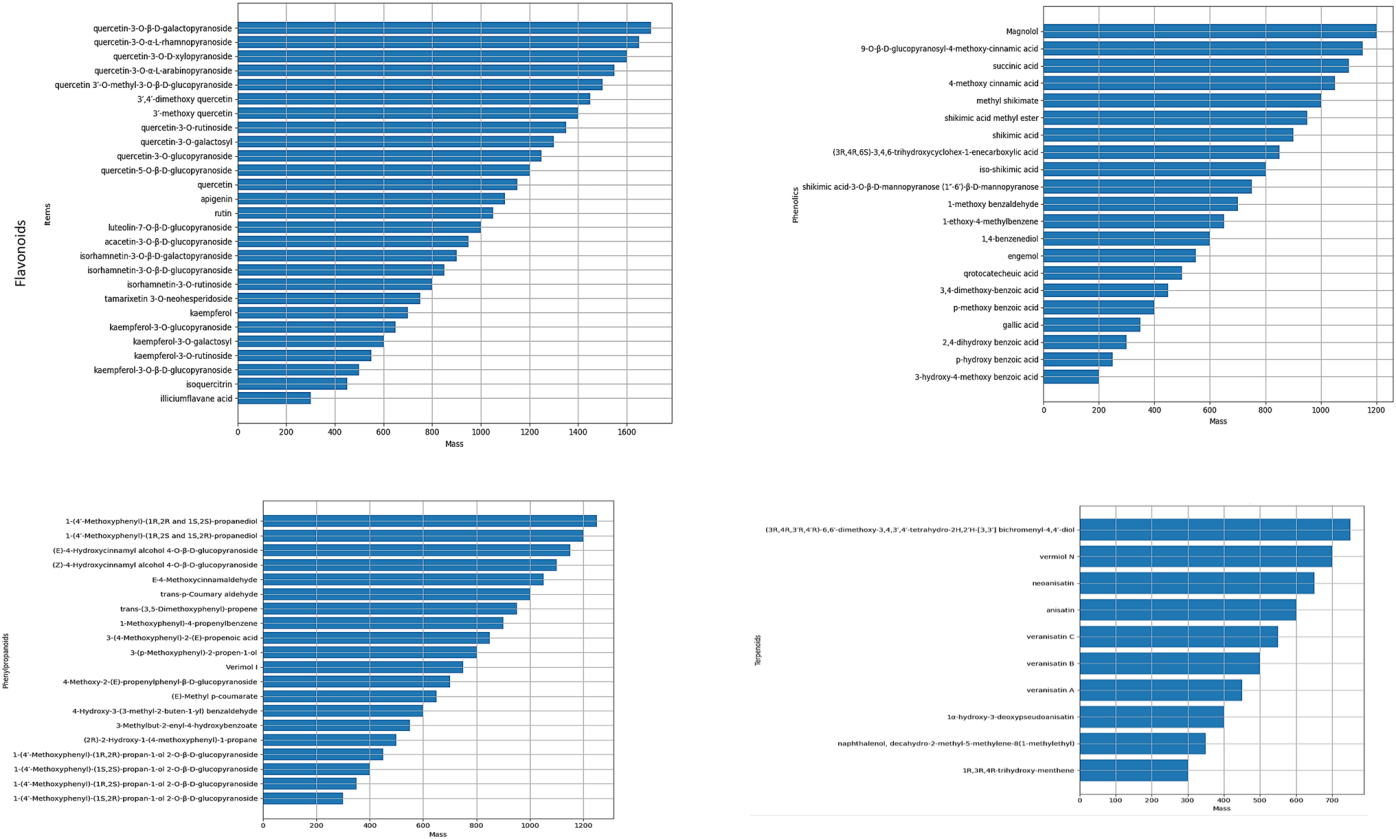
Phytochemicals compounds found in* Illicium verum* extract

*Illicium verum* extract provides total phenolic compounds 5154.0 ± 21.63 (ppm GAE) and total flavonoids 208.5 ± 9.96 (ppm QE). **Figure **1 demonstrates the most common identified compounds from *Illicium verum* fruits extract, that are classified as flavonoids, phenols, phenylpropanoids, and terpenoids. These compounds exhibit strong antioxidant and anti-inflammatory activities, helping to neutralize free radicals, reduce inflammation, and combat infections. These compounds collectively contribute to the diverse pharmacological benefits of star anise, making it a valuable ingredient in both culinary and medicinal applications. Table 1 provides the data relevant to the active compounds used to assess their biological activities and can be further applied as ligands in molecular docking.

**Table 1:** Biological activities of *Illicium verum* compounds “tentative compounds” toward anti-inflammatory and antioxidant simulation

Phenolic & flavonoids compounds	Conc. (μg/g extract)	Mass (g/mol)	Chemical formula		2D structures	Biological process	SMILES	Biological activity (Pa)		
								Anti-inflammatory		Antioxidant
Apigenin-7-glucoside	299.39 ± 5.27	432.38	C_21_H_20_O_10_			Anti-inflammatory, Antioxidant, Anticancer	C1 = CC(= CC = C1C2 = CC(= O)C3 = C(C = C(C = C3O2)O[C@H]4[C@@H]([C@H]([C@@H]([C@H](O4)CO)O)O)O)O)O	0,707**		0,831*
Caffeic	13.15 ± 1.02	180.16	C_9_H_8_O_4_			Antioxidant, Antimicrobial, Anti-inflammatory	C1 = CC(= C(C = C1/C = C/C(= O)O)O)O	0,651		0,603
Cateachin	22.2 ± 1.34	290.27	C_15_H_14_O_6_			Antioxidant, Cardioprotective, Antidiabetic	C1[C@@H]([C@H](OC2 = CC(= CC(= C21)O)O)C3 = CC(= C(C = C3)O)O)O	0,548		0,810*
Chlorogenic	9.3 ± 0.88	354.31	C_16_H_18_O_9_			Antioxidant, Anti-inflammatory, Hepatoprotective	C1[C@H]([C@H]([C@@H](C[C@@]1(C(= O)O)O)OC(= O)/C = C/C2 = CC(= C(C = C2)O)O)O)O	0,598		**0,785**
Chrysin	68.76 ± 1.58	254.2	C_15_H_10_O_4_			Anticancer, Anti-inflammatory, Aromatase inhibitor	COC1 = CC2 = C(C(= C1)OC)C(= O)C = C(O2)C3 = CC = CC = C3	0,589		0,531
Cinnamic	43.22 ± 1.56	148.16	C_9_H_8_O_2_			Antimicrobial, Antioxidant, Antidiabetic	C1 = CC = C(C = C1)/C = C/C(= O)O	0,656		0,489
Coumarin	275.67 ± 2.77	146.14	C_9_H_6_O_2_			Anticoagulant, Antimicrobial, Vasodilator	C1 = CC = C2C(= C1)C = CC(= O)O2	0,615		0,389
Ferulic	56.45 ± 2.37	194.18	C_10_H_10_O_4_			Antioxidant, Neuroprotective, UV-protective	COC1 = C(C = CC(= C1)/C = C/C(= O)O)O	0,604		0,540
Gallic	27.3 ± 1.33	170.12	C_7_H_6_O_5_			Antioxidant, Anticancer, Antimicrobial	C1 = C(C = C(C(= C1O)O)O)C(= O)O	0,548		0,520
Gentisic	10.8 ± 1.05	154.12	C_7_H_6_O_4_			Anti-inflammatory, Antioxidant	C1 = CC(= C(C = C1O)C(= O)O)O	**0,716**		0,406
Kaempferol	70.99 ± 1.33	286.24	C_15_H_10_O_6_			Antioxidant, Anticancer, Cardioprotective, Antiviral	C1 = CC(= CC = C1C2 = C(C(= O)C3 = C(C = C(C = C3O2)O)O)O)O	0,676		0,856*
*p*-coumaric	48.00 ± 1.05	164.16	C_9_H_8_O_3_			Antioxidant, Anti-inflammatory, Antimicrobial	C1 = CC(= CC = C1/C = C/C(= O)O)O	0,641		0,553
*p*-hydroxybenzoic	32.9 ± 1.12	138.12	C_7_H_6_O_3_			Antioxidant, Antimicrobial, Preservative	C1 = CC = C(C = C1)C(C(= O)C2 = CC = C(C = C2)O)O	0,692		0,222
Protocatechuic	344.5 ± 4.58	154.12	C_7_H_6_O_4_			Antioxidant, Antidiabetic, Antiproliferative	C1 = CC(= C(C = C1C(= O)O)O)O	0,692		0,222
Quercetin	130.50 ± 2.05	302.24	C_15_H_10_O_7_			Antioxidant, Antiviral, Anti-inflammatory, Anticancer	C1 = CC(= C(C = C1C2 = C(C(= O)C3 = C(C = C(C = C3O2)O)O)O)O)O	0,689		0,872*
Rosmarinic	415.89 ± 4.97	360.32	C_18_H_16_O_8_			Antioxidant, Anti-inflammatory, Neuroprotective, Antiviral	C1 = CC(= C(C = C1C[C@H](C(= O)O)OC(= O)/C = C/C2 = CC(= C(C = C2)O)O)O)O	0,453		0,539
Rutin	75.18 ± 1.73	610.52	C_27_H_30_O_16_			Antioxidant, Vasoprotective, Anti-inflammatory, Capillary-strengthening agent	C[C@H]1[C@@H]([C@H]([C@H]([C@@H](O1)OC[C@@H]2[C@H]([C@@H]([C@H]([C@@H](O2)OC3 = C(OC4 = CC(= CC(= C4C3 = O)O)O)C5 = CC(= C(C = C5)O)O)O)O)O)O)O)O	0,728**		0,923*
Salycilic	79.77 ± 2.15	138.12	C_7_H_6_O_3_			Analgesic, Anti-inflammatory, Keratolytic	C1 = CC = C(C(= C1)C(= O)O)O	**0,713**		0,318
Sinapic	4.85 ± 0.66	224.22	C_11_H_12_O_5_			Antioxidant, Anti-inflammatory, Hepatoprotective	COC1 = CC(= CC(= C1O)OC)/C = C/C(= O)O	0,612		0,576
Syringic	40.08 ± 1.41	198.18	C_9_H_10_O_5_			Antioxidant, Antimicrobial, Antidiabetic	COC1 = CC(= CC(= C1O)OC)C(= O)O	0,498		0,403
Vanillic	12.78 ± 1.27	168.16	C_8_H_8_O_3_			Antioxidant, Neuroprotective, Anti-inflammatory	COC1 = C(C = CC(= C1)C(= O)O)O	0,505		0,374
Volatile compounds	**% Area**									
Aromadendrene	**1.68**	204.36	C_15_H_24_			Anti-inflammatory, Antibacterial, Antifungal	CC1CCC2C1C3C(C3(C)C)CCC2 = C	0,318	**-**	
Camphene	0.44	136.24	C_10_H_16_			Bronchodilator, Antioxidant, Antimicrobial	CC1(C2CCC(C2)C1 = C)C	0,245	-	
Caryophyllene oxide	0.23	220.36	C_15_H_24_O			Anti-inflammatory, Antitumor, Antifungal	C[C@@]12CC[C@@H]3[C@H](CC3(C)C)C(= C)CC[C@H]1O2	**0,759**	0,144	
*cis*- Anethole	**2.26**	148.2	C_10_H_12_O			Estrogenic, Antioxidant, Antispasmodic	C/C = C\C1 = CC = C(C = C1)OC	0,526	0,324	
Elemol	0.49	222.36	C_15_H_26_O			Antimicrobial, Antioxidant, Anti-inflammatory	CC(= C)[C@@H]1C[C@@H](CC[C@@]1(C)C = C)C(C)(C)O	0,793*	0,168	
Estragole	**14.4**	148.2	C_10_H_12_O			Estrogenic, Antibacterial, Neurotoxic (at high doses)	COC1 = CC = C(C = C1)CC = C	0,448	0,337	
Farnesol	0.5	222.36	C_15_H_26_O			Antioxidant, Skin conditioning, Anti-inflammatory	CC(= CCCC(= CCCC(= CCO)C)C)C	0,643	0,549	
Foeniculin	**8.86**	148.2	C_10_H_12_O			Spasmolytic, Mild antimicrobial activity	C/C = C/C1 = CC = C(C = C1)OCC = C(C)C	0,548	0,482	
Germacrene B	0.91	204.36	C_15_H_24_			Antitumor, Antibacterial, Insecticidal	C/C/1 = C\CC/C(= C/CC(= C(C)C)CC1)/C	0,526	-	
Linalool	**1.92**	154.26	C_10_H_18_O			Anti-inflammatory, Sedative, Anxiolytic	CC(= CCCC(C)(C = C)O)C	0,537	0,380	
Linalool acetate	**1.96**	196.28	C_12_H_20_O_2_			Sedative, Antispasmodic, Antimicrobial	CC(= CCCC(C)(C = C)OC(= O)C)C	**0,751**	0,389	
Safrole	0.21	178.18	C_10_H_10_O_2_			Antimicrobial, Hepatotoxic, Carcinogenic (at high doses)	C = CCC1 = CC2 = C(C = C1)OCO2	0,309	0,297	
Spathulenol	0.27	220.36	C_15_H_24_O			Anti-inflammatory, Antioxidant, Antifungal	C[C@@]1(CC[C@@H]2[C@@H]1[C@H]3[C@H](C3(C)C)CCC2 = C)O	0,521	-	
*trans* – Anethole	**47.16**	148.2	C_10_H_12_O			Antispasmodic, Antimicrobial, Flavoring agent	C/C = C/C1 = CC = C(C = C1)OC	0,526	0,324	
*trans* – Nerolidol	**1.01**	222.36	C_15_H_26_O			Antifungal, Antioxidant, Anti-inflammatory	CC(= CCC/C(= C/CCC(C)(C = C)O)/C)C	0,800*	0,431	
*trans*- *α*- Bergamotene	**1.42**	204.36	C_15_H_24_			Anti-inflammatory, Insecticidal, Antitumor	CC1 = CC[C@H]2C[C@@H]1[C@]2(C)CCC = C(C)C	0,614	0,319	
*trans*- *α*- Bergamotene	0.33	220.36	C_15_H_26_O			Antioxidant, Antimicrobial, Hepatoprotective	CC1 = CC[C@H]2C[C@@H]1[C@]2(C)CCC = C(C)C	0,566	0,319	
*α*- Cadinol	0.55	136.24	C_15_H_24_			Insecticidal, Antioxidant, Antifungal	CC1 = C[C@H]2[C@@H](CC[C@@]([C@@H]2CC1)(C)O)C(C)C	0,476	-	
*α*- Copaene	0.37	136.24	C_10_H_16_			Antimicrobial, Anti-inflammatory, Bronchodilator	CC1 = CC[C@H]2[C@H]3[C@@H]1[C@@]2(CC[C@H]3C(C)C)C	0,490	-	
*α*-Pinene	**2.48**	204.36	C_10_H_16_			Antimicrobial, Antiviral	CC1 = CCC2CC1C2(C)C	0,490	**-**	
*α*-Thujene	0.43	136.24	C_15_H_24_			Anti-inflammatory, Antimicrobial, Antitumor	CC1 = CCC2(C1C2)C(C)C	0,807*	-	
*β* – Bisabolene	**1.12**	204.36	C_10_H_16_			Anti-inflammatory, Antibacterial, Antiviral	CC1 = CC[C@H](CC1)C(= C)CCC = C(C)C	**0,726**	0,257	
*β* – Pinene	0.28	136.24	C_15_H_24_			Anti-inflammatory, Antitumor, Antimicrobial	CC1(C2CCC(= C)C1C2)C	0,611	-	
*β* –Caryophyllene	**1.24**	204.36	C_10_H_16_			Antioxidant, Antimicrobial, Anti-inflammatory	C/C/1 = C\CCC(= C)[C@H]2CC([C@@H]2CC1)(C)C	**0,745**	0,174	
*β* –Myrcene	0.27	204.36	C_15_H_24_			Antifungal, Insecticidal, Anti-inflammatory	CC(= CCCC(= C)C = C)C	0,388	0,470	
*μ*- Cadinene	0.7	204.36	C_15_H_24_			Anti-inflammatory, Antibacterial, Antifungal	C[C@H]1CC[C@H]([C@H]2[C@H]1C = C[C@@H](C2)C)C(C)C	0,327	-	

Pa > 0.7: high probability; 0.5 < Pa ≤ 0.7: moderate probability. * identified as a ligand based on their Hight biological activity score (top 4 high probability/mediator); ** identified as a ligand based on high probability for both mediators (2 ligands); mass of tentative compounds computed by PubChem 2.1 (PubChem release 2021.05.07). Chemical formulas, 2D structures & SMILES obtained from OPSIN©; SMILES, computed by OEChem 2.3.0 (PubChem release 2025.04.14)

### *B. alexandrina* proteins (macromolecules) and biological/molecular pathway outcomes

**Fig. 2:**
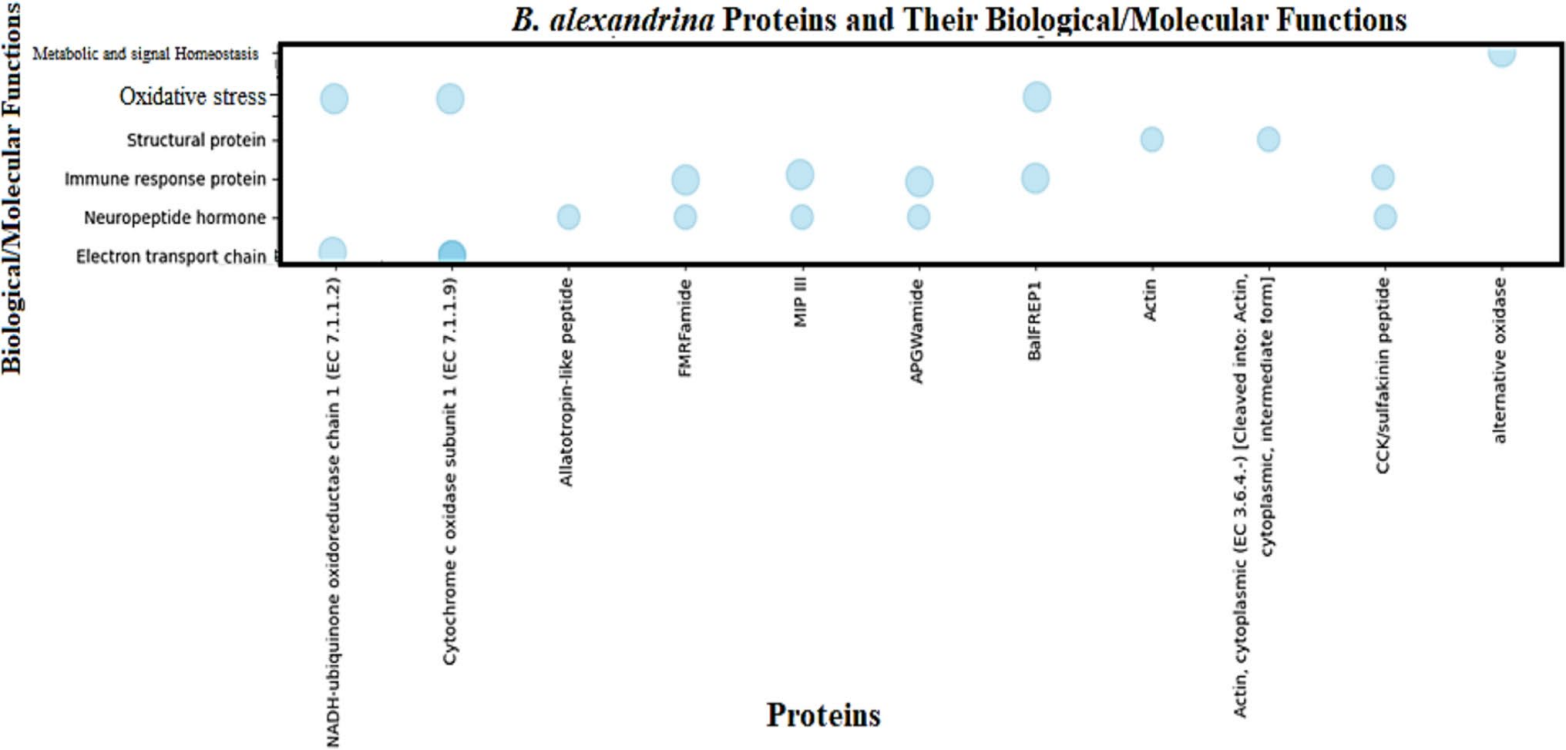
Visually representative bubble chart of *B. alexandrina* proteins and their associated functions. The size of each bubble indicates the length of the function description

*B. alexandrina* proteins play crucial roles in various biological and molecular pathways, impacting the snail’s physiology and its immune interaction. Bubble chart (Fig. 2) provides a visually appealing representation of the proteins and their functions, with the size of each bubble indicating the length of the function description.

Table 2 demonstrates structural validation and characterization of *B. alexandrina* proteins for molecular docking analysis. Ramachandran plot analysis of the selected *Biomphalaria alexandrina* proteins confirmed that the majority of the residues fell within favored and allowed regions, indicating good stereochemical quality and reliable backbone geometry. Only a few outlier residues were observed, suggesting minimal structural anomalies and validating the suitability of the modeled proteins for subsequent molecular docking and simulation studies.

**Table 2:** Structural validation and characterization of Biomphalaria alexandrina proteins for molecular docking analysis

ACCESSION**	Entry Name*** Protein names	Length aa**	Mass**	Gene Names	3D structure	Ramachandran plot
AAV35424	Q5C9L0_BIOAL NADH-ubiquinone oxidoreductase chain 1 (EC 7.1.1.2)*	148	16,821	ND1		
AAZ41263	Q2QFN7_BIOAL Cytochrome c oxidase subunit 1 (EC 7.1.1.9) *	138	15,288	COI		
AAZ41263	Q2QFQ9_BIOAL Cytochrome c oxidase subunit 1 (EC 7.1.1.9)	217	23,743	COI		
AAG30333	Q9GAB5_BIOAL Cytochrome c oxidase subunit 1 (EC 7.1.1.9)	193	20,977	COI		
AGV39323	W5RFW7_BIOAL Cytochrome c oxidase subunit 1 (EC 7.1.1.9)	214	23,403			
ATN96645	A0A2D1GUG5_BIOAL Allatotropin-like peptide*	129	14,341			
QCX41799	A0A4Y5QRP7_BIOAL FMRFamide*	292	34,576			
QCX41800	A0A4Y5QRZ2_BIOAL MIP III *	119	12,985	MIP III		
QCX41798	A0A4Y5QS42_BIOAL APGWamide*	206	22,593	APGWamide		
AAC47702	O18539_BIOAL BalFREP1*	118	14,004	BalFREP1	-	
AAN75105	Q8I9W2_BIOAL Actin*	174	19,642	Q8I9W2		
AAK68711	ACTC_BIOAL Actin, cytoplasmic (EC 3.6.4.-) [Cleaved into: Actin, cytoplasmic, intermediate form]	376	41,911	ACTC		
WKK27864	CCK/sulfakinin peptide	138	58	CCK, SK		
UNU90931	alternative oxidase	342	40 kDa	AOX		

* identified as macromolecules for further molecular docking; ** data obtained from PubChem@NCBI; *** data obtained from UniProt & GO annotation databases; 3D structure obtained from AlphaFold Protein Structure Database

Key proteins related to their corresponding biological/molecular functions, cellular components, and biological activity pathways, which are illustrated via GO annotation databases, are listed in Table 3 using the UniProt database. The key feature of protein activities includes essential proteins for the electron transport chain; these proteins are involved in cellular respiration and energy production, such as NADH-ubiquinone oxidoreductase chain 1 and cytochrome c oxidase subunit 1. Neuropeptides, such as allatotropin-like peptide, FMRFamide, macrophage inflammatory protein (MIP III), and APGWamide, regulate various physiological processes, including muscle contraction and neurotransmission. Furthermore, immune response proteins, such as BalFREP1, which is crucial for maintaining cell shape, motility, and division, including actin proteins, and alternative oxidase proteins that provide alternative pathways for electron transport, help organisms manage oxidative stress, play a role in snail defense mechanisms against pathogens.

**Table 3:** Functional annotation and gene ontology mapping of *Biomphalaria alexandrina* target proteins

GO code *** Protein names	Biological process***	Ancestor biological Pathway Gene Ontology (biological process/ molecular function)***	Gene Ontology (cellular component) ***
GO:0008137 NADH-ubiquinone oxidoreductase chain 1 (EC 7.1.1.2)*	generation of precursor metabolites and energy transmembrane transport	aerobic respiration [GO:0009060] NADH dehydrogenase (ubiquinone) activity [GO:0008137]; NADH dehydrogenase activity [GO:0003954]	mitochondrial inner membrane [GO:0005743]
GO:0004129 Cytochrome c oxidase subunit 1 (EC 7.1.1.9) *	generation of precursor metabolites and energy transmembrane transport	electron transport coupled proton transport [GO:0015990]; mitochondrial electron transport, cytochrome c to oxygen [GO:0006123] cytochrome-c oxidase activity [GO:0004129]; heme binding [GO:0020037]; metal ion binding [GO:0046872]	mitochondrial inner membrane [GO:0005743]
GO:0004129 **Cytochrome c oxidase subunit 1 (EC 7.1.1.9)**			mitochondrial inner membrane [GO:0005743]
GO:0004129 **Cytochrome c oxidase subunit 1 (EC 7.1.1.9)**			mitochondrial inner membrane [GO:0005743]
GO:0004129 **Cytochrome c oxidase subunit 1 (EC 7.1.1.9)**			mitochondrial inner membrane [GO:0005743]
GO:0016020 Allatotropin-like peptide*	Regulating Development Metabolism Reproductive Functions		membrane [GO:0016020]
GO:0007218 FMRFamide*	Signaling	neuropeptide signaling pathway [GO:0007218]	extracellular region [GO:0005576]
GO:0005179 MIP III *	• Neuropeptide signaling pathway • Regulation of muscle contraction • Modulation of neurotransmission	hormone activity [GO:0005179]	extracellular region [GO:0005576]; transport vesicle [GO:0030133]
GO:0005184 APGWamide*	• Neuropeptide signaling pathway • Regulation of muscle contraction • Modulation of neurotransmission	GO:0005184 (neuropeptide hormone activity) GO:0007268 (synaptic transmission)	• Extracellular region • Secreted
**O18539_BIOAL** BalFREP1*	• Immune response Pathogen recognition • Defense response	GO:0006955 (immune response) GO:0006952 (defense response) GO:0002376 (immune system process)	collagen-containing extracellular matrix [GO:0062023]; extracellular space [GO:0005615]
GO:0016787 Actin*	Cell motility Muscle contraction Cell division Maintenance of cell shape Intracellular transport	hydrolase activity [GO:0016787]	
GO:0005524 **Actin, cytoplasmic (EC 3.6.4.-) [Cleaved into: Actin, cytoplasmic, intermediate form]**		ATP binding [GO:0005524]; hydrolase activity [GO:0016787]	cytoplasm [GO:0005737]; cytoskeleton [GO:0005856]
GO:0005179 **CCK/sulfakinin peptide**	Regulation of feeding, digestion, and various physiological processes	GO:0005179 (hormone activity), GO:0005184 (neuropeptide hormone activity)	Extracellular region, secreted
GO:0009916 **Alternative oxidase**	Stress Response Pathogen Resistance Energy Production	GO:0009916 (alternative oxidase activity), GO:0005739 (mitochondrion)	Mitochondrial inner membrane

* identified as macromolecules for further molecular docking; ** data obtained from PubChem@NCBI; *** data obtained from UniProt & GO annotation databases; 3D structure obtained from AlphaFold Protein Structure Database; Ancestor Pathway obtained from https://www.ebi.ac.uk/QuickGO/term/; aa, amino acid

These proteins contribute to the overall health of snails, their ability to respond to environmental stressors, and their interactions with pathogens to activate the immune response, making them vital for understanding the biological and molecular mechanisms of *B. alexandrina*.

### Molecular docking outcomes

This study is based on the selection of specific proteins from *B. alexandrina* for molecular docking studies because of their biological relevance to vital physiological functions and their potential as molecular targets for oxidative/immune and molluscicidal activities. Disruption of these proteins could lead to biological alterations in snails, making them strategic targets for controlling *B. alexandrina* populations. This choice reflects a targeted approach to identify natural compounds from *Illicium verum* that can interfere with essential snail functions, thus supporting the development of environmentally safe biocontrol agents.

Based on the molecular docking results, the bioactive compounds from *Illicium verum* demonstrated strong binding affinities with key proteins of *B. alexandrina*, supporting their potential biological relevance. Notably, kaempferol, quercetin, and rutin presented the highest binding scores across multiple protein targets, including cytochrome c oxidase subunit 1, FMFamide, and BaFREP1. These proteins are critical to mitochondrial respiration, neuromodulation, immune defense, and cellular structure, suggesting that these compounds may disrupt essential physiological functions in the snail host.

**Fig. 3:**
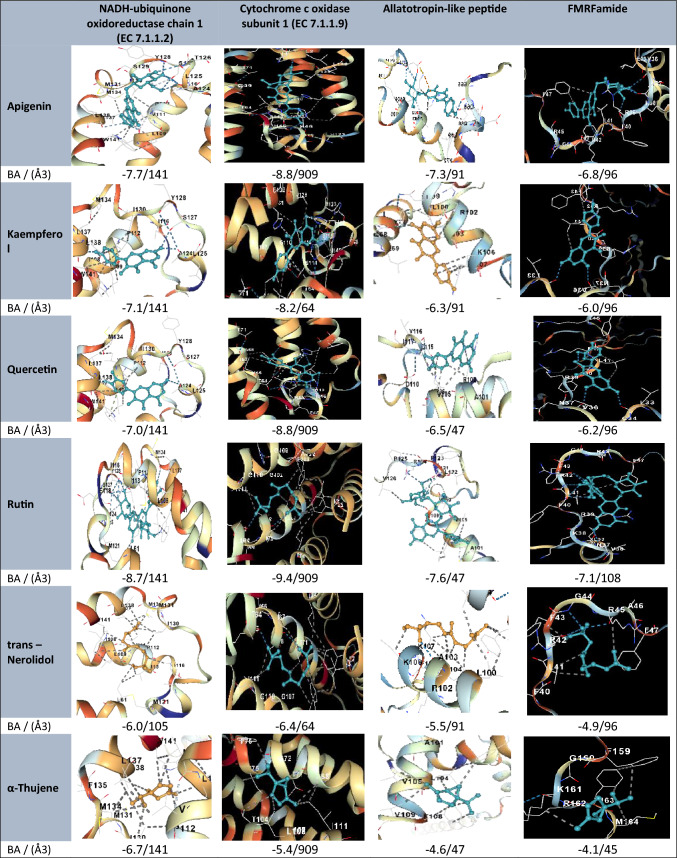

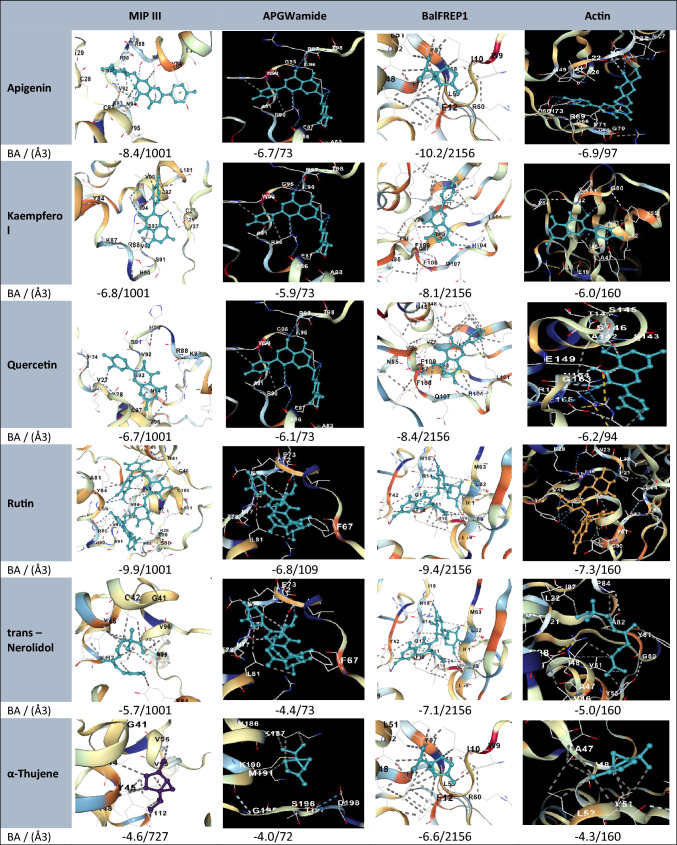
Molecular docking and binding affinity visualization of star anise ligands with *B*. *alexandrina* proteins. BA, Vina score (binding affinity in kcal/mol); (Å3), cavity volume

The molecular docking results revealed that selected phytochemicals from Illicium verum exhibited strong binding affinities with key *B. alexandrina* target proteins. Notably, compounds such as kaempferol and quercetin presented the lowest binding energy scores (e.g. − 7.6 to − 9.4 kcal/mol), indicating stable and favorable interactions. These top-performing ligands engage in multiple hydrogen bonds and hydrophobic interactions, particularly in proteins associated with oxidative stress and immune modulation. The results support the potential of these compounds as bioactive agents with molluscicidal or immunomodulatory effects. Figure 3 confirmed the consistent interactions, and 3D visualization with CB-Dock2 reinforced the specificity and stability of the ligand‒protein complexes. Collectively, the docking outcomes align well with the observed in vivo effects and support the pharmacological potential of *I. verum* as a source of molluscicidal and immunomodulatory agents.

The validity of the native ligand docking results is further supported by the use of CB-Dock2 as an advanced cavity-detection-guided docking platform that enhances precision by identifying optimal binding pockets prior to docking. Currently, CB-Dock2 successfully redocked the native ligands into the most probable active sites of the selected B. *alexandrina* proteins, confirming that the docking process accurately replicated the native binding conformation. Moreover, CB-Dock2’s integrated scoring function provided consistent binding affinity estimates, further validating the docking reliability. These outcomes substantiate that the docking protocol, including pocket prediction and scoring, is robust and suitable for evaluating the interaction of *Illicium verum* phytochemicals with functionally significant snail proteins.

### PICKLE database interaction outcomes

**Fig. 4:**
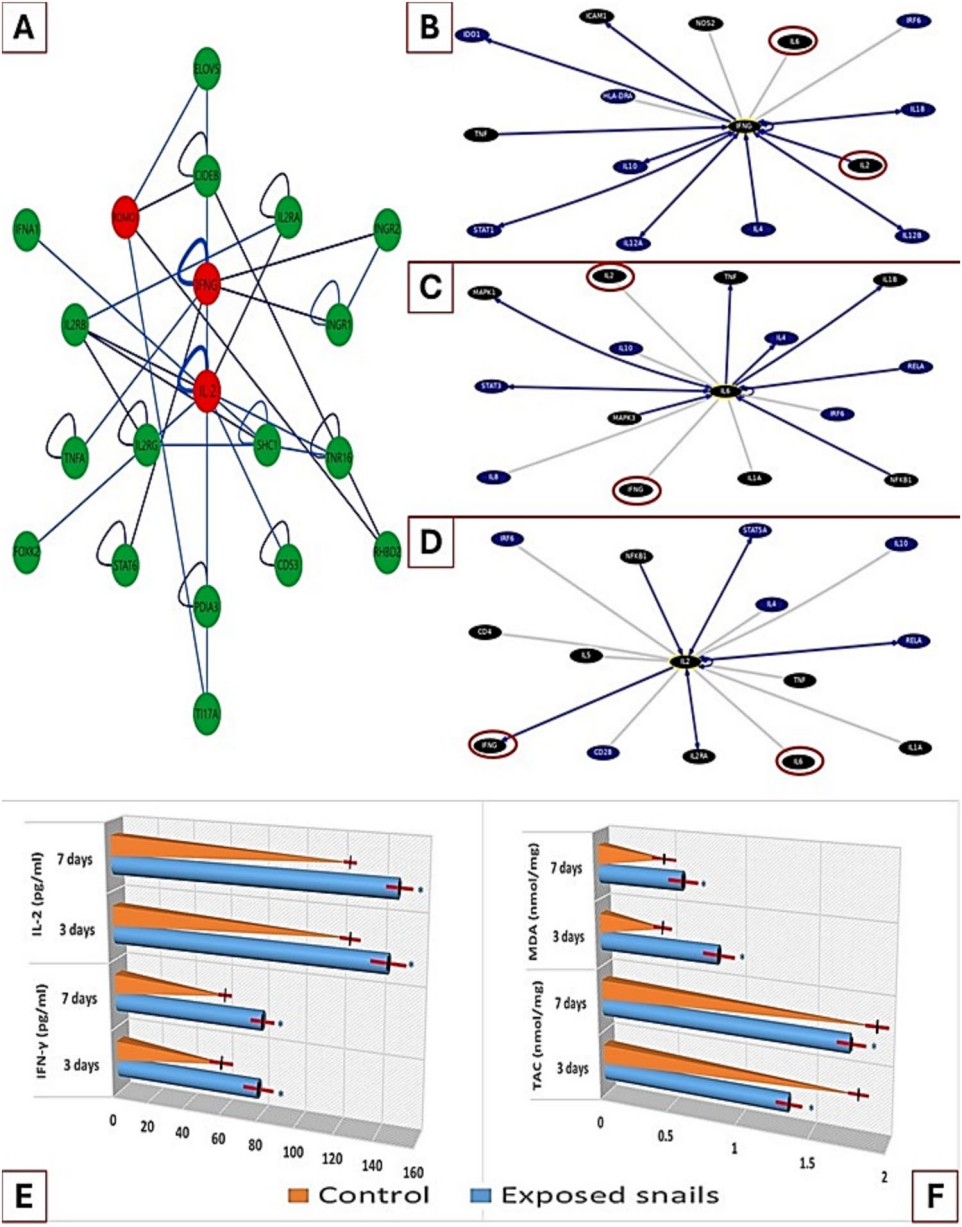
**I.** In silico simulation of oxidative and inflammatory protein/gene interactions. **A:** Concentric layout network visualization of the inflammatory and oxidative biomarkers. Data obtained from PICKLE (with 30 edges and 58 nodes, PPI quality 15), the cross-checking (default) filtering method, normalization level protein (UniProt), first neighbors and any interactions between them network setup). (Accessed on 3 March 2024). **B-D:** Top 15 interacting genes of IFN-γ, IL-2, and IL-6. The data were retrieved from the UCSC Genome Browser Gene Interaction Graph, highlighting the Drug Bank interactions obtained through gene interactions and pathways. Accessed on 18 May 2024. The interactions are colored by support. Genes with black colored: treatment hits by Drug Bank; gray: interactions from several datasets with only text mining; light blue: interaction database; blue: pathway database. ROMO1: reactive oxygen species modulator; IFNG: interferon gamma; IL-2: interleukin-2; IL-6: interleukin-6. **II:** Effects of Illicium verum on oxidative/inflammatory biomarkers in control and exposed snails. **E:** Anti-inflammatory capacity (pg/ml) of snail hemolymph. **F:** antioxidant capacity nmol/g of snail tissue homogenate. The values are presented as mean ± standard deviation (SD) after 3- and 7-day time intervals; *n* = 10 snails. *IFN-γ* interferon gamma, *IL-2* interleukin-2, *TAC* total antioxidant capacity, *MDA* malondialdehyde. *p ≤ 0.05 vs control group

The interactions of the studied inflammatory cytokines and oxidative (ROMO1; reactive oxygen species modulator) mediators with other genes as well as with each other are depicted in Fig. 4A and were retrieved from the PICKLE database. The interactions among ROMO1, IFN-γ, and IL-2 involve active interaction and complex signaling pathways that play crucial roles in the immune response. ROS modulators can influence the balance of oxidative stress and antioxidant defense, affecting various cellular processes. The diagrammatic representation demonstrates the involvement of IFN-γ in pathways related to the activation of macrophages and the enhancement of antigen presentation, leading to increased ROS production. IL-2 promotes T-cell proliferation and differentiation. Together, these interactions modulate immune cell function and inflammatory responses, impacting overall immune regulation and potential therapeutic interventions for various pathological conditions.

### Gene–gene interactions and pathway outcomes

The gene‒gene interactions and pathways associated with the inflammatory biomarkers IFN-γ, IL-2, and IL-6 were derived from curated databases and text mining (Fig. 4B, C, D) at the UCSC Genomics Institute. These interactions and pathways illustrate the complex regulatory network governing immune responses, highlighting the importance of these cytokines signaling in health and disease.

The diagrammatic representations of the IFN-γ-, IL-2-, and IL-6-interacting pathways highlight their impacts on activating the JAK-STAT signaling cascade, which translocate to the nucleus and induces the expression of various genes, including those involved in the inflammatory pathway. Interferon regulatory factor (IRF) interactions, which regulate genes involved in immune responses, are the same.

IFN-γ is a critical regulator of inducible nitric oxide (iNOS) and NO production, which induces their expression by promoting the transcription of the NOS2 gene (which encodes iNOS). This interaction is crucial for host defense against infections and the modulation of immune and inflammatory responses.

IL-2 and IL-6 interact with the mitogen-activated protein kinase (MAPK) pathway, and activation of the MAPK pathway leads to the transcription of genes involved in cell cycle progression. Additionally, the interaction between IL-6 and NF-κB is a critical axis in the regulation of cellular, immune, and inflammatory responses.

IFN-γ, IL-2 and IL-6 can have feedback effects on their own production and the production of other cytokines, creating a complex network of regulatory effects. Proinflammatory and anti-inflammatory signals are balanced to maintain immune homeostasis. While this pathway is essential for normal immune function, its dysregulation can contribute to the pathogenesis of various inflammatory and immune diseases.

### Immunomodulatory activity

The dry powder of the *Illicium verum* plant (Star anise) as water suspension has acceptable molluscicidal activity, with LC10 of 315 ppm. Therefore, dry plant powder was selected to evaluate its immunomodulatory impact on *B. alexandrina* snails.

The concentrations (pg/ml) of IFN-γ and IL-2 were significantly elevated in the snails exposed to star anise compared to the control group after 3 and 7 days. IFN-γ concentrations in the snails exposed group slightly increased after 7 days than 3 days of exposure as mean values 80.6 ± 2.31 and 80.3 ± 2.85, which were significantly greater (*P ≤ 0.05) than those in the control group (32.7 ± 1.46 and 34.03 ± 1.71), respectively. Il-2 concentrations also showed highest values in the snails in the exposed group after 7 days, with mean representation (72.7 ± 3.59 and 72.3 ± 2.83) and a significant increase (*P ≤ 0.05) compared to the control group (24.8 ± 1.67 and 23.6 ± 1.54) after 3 & 7 days, respectively. The data are summarized in Fig. 4E.

### Oxidative activity

The present data of the effects of *Illicium verum* on the total antioxidant capacity and malondialdehyde in* B. alexandrina* snail’s tissue homogenate indicated significantly decreased (*P ≤ 0.05) TAC activity (0.96 ± 0.06 and 0.77 ± 0.05) expression nmol/g; while, malondialdehyde expressed nmol/g significant increased (*P ≤ 0.05) levels (7.2 ± 0.4 and 8.5 ± 0.5) compared to control group (TAC, 1.3 ± 0.2 and 1.4 ± 0.1; MDA; 6.6 ± 0.2 and 6.3 ± 0.2) after 3 and 7 days of exposure, respectively (Fig. 4F).

### Hemocyte alteration outcomes

**Fig. 5:**
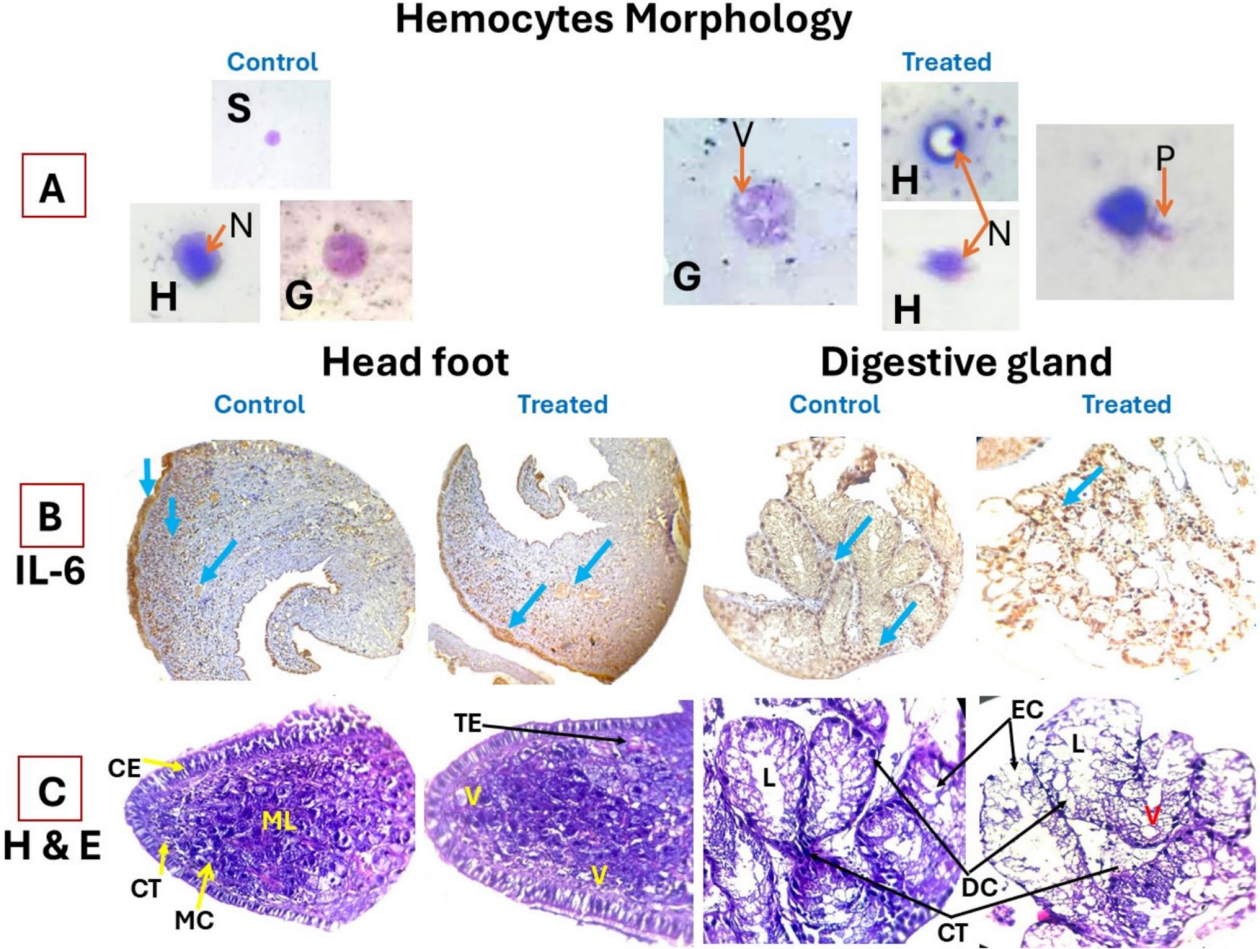
Hemocyte morphology and immunohistochemical/histological investigation of the impact of *illicium verum* on *B. alexandrina* snails (head foot and digestive gland). **A:** Photomicrographs showing snail hemocytes (× 40) of normal control and after exposure to star anise. S, round small hemocyte; H, hyalinocyte; G, granulocyte; N, nucleus; V, vacuoles; P, pseudododia. B: Immunohistochemical changes in IL-6 expression (light blue arrow); normal distribution within the control group and lower expression *B. alexandrina* snail treated with *I. verum.* **C:** Histological changes; control group, normal head foot of *B. alexandrina* snail with connective tissue **(CT),** muscle layer **(ML),** mucous cell **(MC**), and columnar epithelia **(CE)**. Head-foot of *B. alexandrina* snails treated with *I. verum* with muscle fiber degeneration and the presence of empty spaces or vacuoles within the muscle tissue **(V)** and connective tissues with edema and densely stained in outer layer **(TE**). Normal digestive gland of the control group contained lumen **(L)**, excretory cell **(EC),** digestive cell **(DC)**, and connective tissue among hepatopancreatic tubules **(CT)**. The digestive glands of *B. alexandrina* snails treated with a sublethal dose of *I. verum* caused necrotic changes in excretory cells **(EC)** and digestive cells **(DC)**, dilation of the lumen, fusion of more than two tubules into a larger lumen **(L**), connective tissue among tubules (**CT)** and cellular vacuoles (**V**) were observed

The current morphological investigation identified one small hemocyte type and two types of larger hemocytes, hyalinocytes and granulocytes (Fig. 5A). The exposure of *B. alexandrina* to *I.* verum resulted in an increasing number of hemocyte populations with distinct morphological changes. Granulocytes displayed increased granularity and vacuolization, along with irregularities in the cell membrane and the emergence of pseudopodia. Moreover, hyalinocytes exhibited nuclear condensation, disrupted membrane integrity, and occasional extension of pseudopodia.

### Immunohistochemical results

*Illicium verum* treatment effectively reduces IL-6 expression (Fig. 5B), highlighting its potential as a natural anti-inflammatory compound. Comparing treated and untreated control snails, the head food of those exposed to the anise extract showed a marked reduction in IL-6 tissue expression, with estimated percentages of positive morphometric studies of 2.1% compared with 5.89% in the control group. Also, the digestive system of the treated group presented a lower expression, with a positive estimated percentage of 1.41%, than the control group did (4.46%).

### Histological results

The histological changes in the head-foot, and digestive glands of *B. alexandrina* are demonstrated in Fig. 5C. In the typical head-foot region, the outer cuticular layer acts as a protective covering for the foot. A tall columnar epithelium with basal nuclei in its cells is located within this lining. Unicellular glands, located within the modified sac-like cells of the columnar epithelium, open externally through the cuticular layer to the foot surface. These monocellular glands are responsible for mucus secretion. Transverse and longitudinal muscle fibers are interspersed between them. Oblique muscle fibers are densely packed and make up a significant portion of the foot muscles**.** The *B. alexandrina* snails’ head feet treated with *I. verum* induced histopathological alterations characterized by muscle fiber degeneration, the formation of empty spaces or vacuoles, and the accumulation of toxic agents in the tested plant extracts**.**

The digestive gland of *B. alexandrina*, also referred to hepatopancreas, is almost similar in their basic histological structure. It is a complex tubular gland surrounded by connective tissue lined with simple epithelium throughout its tubules, which are arranged around a tiny, atypical lumen. These cells mostly include two different cell types: excretory cells, which contain granular cytoplasm, and digestive cells, which are columnar with basal nuclei. Histological changes in the digestive gland were observed after treatment with *I. verum*. Notably, the response of digestive gland tissue did not vary greatly from one snail to the others. Nevertheless, certain tubules within the same digestive gland exhibited more pronounced effects compared to others. The connective tissues of the digestive gland displayed histological abnormalities that led to the degradation of all the cells, resulting in enlarged lumens of the tubules. The rupture of epithelial cells led to the loss of cell boundaries and the diffusion of cellular contents throughout the tissue.

## Discussion

Star anise possesses antioxidant, anti-inflammatory, and antimicrobial properties that support sustainable, eco-friendly applications in health and agriculture. Given the role of snail immunity and environmental factors in schistosome infection, exploring the immunological and histological effects of star anise offers a promising, environmentally conscious strategy for molluscicidal control. When integrated with computational tools and molecular docking, these investigations allow precise prediction of tentative compound–protein interactions, helping identify key molecular targets and optimize star anise’s therapeutic potential against disease-transmitting snails.

In silico applications have become an indispensable tool in modern drug discovery, offering rapid and cost-effective means to predict the biological activity, stability, and binding efficiency of potential therapeutic compounds [33]. The validity of the native ligand docking was confirmed using CB-Dock2, which accurately redocked the ligands into the predicted active sites, indicating close alignment with the crystallographic poses. These findings support the reliability of the docking protocol and scoring function for assessing *Illicium verum* compound interactions with target proteins.

The results of the present study indicate that the interaction between the proteins of *B. alexandrina* and the bioactive compounds in star anise involves a dynamic interplay that affects various physiological and immunological functions. Antioxidant and anti-inflammatory compounds from star anise can stabilize and modulate proteins involved in the immune response, mitochondrial function, neurotransmission, and cellular structure [34, 35]. The promising bioactivities and low cytotoxicity of synthetic compounds [36] highlight the potential of structurally diverse natural and semisynthetic molecules, as demonstrated in our study with *I. verum* phytocompounds, which similarly exhibited strong binding affinities and favorable interaction profiles in molecular docking simulations, supporting their role as prospective antiparasitic and antimicrobial agents. This intricate crosstalk highlights the potential of star anise as a therapeutic agent for managing oxidative stress and inflammation in these snails.

The current phytochemical analysis of star anise indicates highly valuable compounds, including kaempferol, which is an antioxidant and anti-inflammatory agent [37], can potentially stabilize proteins such as cytochrome c oxidase subunit 1 and the NADH-ubiquinone oxidoreductase chain BalFREP1 and MIP III. p-Hydroxybenzoic acid modulates immune responses, potentially affecting inflammatory proteins [38]. Quercetin is an anti-inflammatory and antioxidant, likely impacting protein mediators of these interactions [39]. These proteins can potentially stabilize proteins such as Actin [40] and MIP III, which might modulate their expression and activity, affecting immune responses [41]. All of these active compounds and their interactions are the main causes of the logical interpretation of the current further biological outcomes.

The current protein‒ligand complex images shown were selected based on the best binding affinity scores obtained from the AutoDock Vina and CB-Dock2 docking outputs. Specifically, the interactions visualized correspond to the top-ranked binding pose, which represents the lowest binding energy and therefore the most thermodynamically favorable configuration. This selection reflects the standard practice in molecular docking studies, where the pose with the strongest predicted interaction is highlighted for detailed interaction profiling.

The current findings indicate that in accordance with the WHO’s [42] recommendations on plant molluscicides, dry star anise powder has a detrimental effect on *B. alexandrina* snails. Antioxidant, antibacterial, antifungal, antinociceptive, anti-inflammatory, anthelmintic, insecticidal, secretolytic, gastroprotective, sedative, expectorant, spasmolytic, and estrogenic actions are only a few of the potentials that star anise possesses [43]. Our results concerning the biological activity of star anise against *B. alexandrina* are compatible with the results of Shobana & Naidu [44], who reported that star anise is among several spices known to contain bioactive components, including various phenolic and flavonoid compounds. These compounds contribute to its antioxidant, preservative, antibacterial, and antimicrobial properties. Kim et al. [45] reported that the star anise targets the mitochondrial oxidative stress defense mechanism to suppress fungal development.

The current relatively high levels of both phenolics and flavonoids support the observed antioxidant and bioactive potential of the extract, as these compounds are known for their roles in scavenging free radicals and modulating biological pathways. These data are supported by those of Prinsa et al. [46] and Saha et al. [47], who stated that flavonoids, known for their broad spectrum of biological activities, have shown promising interaction profiles in molecular docking studies, underscoring their potential as therapeutic agents for targeting disease-related proteins.

The internal defense systems of invertebrates depend on the intrinsic immune system, which includes both humoral and cellular effectors [48]. Hemocytes are diverse cell types that are essential for defense, operate as the main defense against invasive parasites and bacteria, and acquire different compounds, such as molluscicides, toxic metals, and pesticides, resulting in a cell-mediated immune response [49].

Cytokines are humoral components that are produced as a result of the activity of these cells. IFN‐γ is a cytokine that is crucial for triggering and controlling a variety of immunological reactions, whereas interleukin-2 is a cytokine that represents a key player in the cell-mediated immune response. In this study, the altered concentrations of cytokines may be due to changes in hemocytes count of the exposed groups to modulate the immune response against star anise exposure and mediate phagocytic activity; hence, hemocytes are phagocytic cells that are capable of encapsulating large particles [50]. Additionally, several studies have demonstrated that the percentages of various hemocyte types in aquatic snails changed as a result of medication treatment [27]. Furthermore, a study reported that they are primarily in charge of healing wounds, which call for aggregation at the site of the injury [51], and that they are immunologically functioning cells noticed primarily in snail hemolymph rather than remaining in damaged tissue to respond to external triggers [52]. According to Corrˆea el al. [53], the ability of the internal defense system’s hemocytes and soluble hemolymph components to recognize and kill invading components has been ascribed to the mollusks’ susceptibility level.

The current cytokines/hemocytes interplay can play a role in immune regulation and modulation activities to maintain the resulting effect of star anise on snails. This finding is supported by Iwuozo et al. [54]. Hence, IFN-γ plays significant biological roles in organisms, particularly as a major regulator of host defense processes involved in the phagocytosis of foreign agents [55]. IL-2, which is crucial for immune system function, particularly in mediating immune cell activation and maintenance, was also significantly elevated by star antise administration in the present study. This observation aligns with findings of Iwuozo et al. [54].

Attia et al. [9] revealed that proinflammatory cytokines were elevated during treatment with the star anise in goldfish; these findings are similar to those reported by Tu et al. [56] and Abd El-Hack et al. [57], they supported the result of star anise was considered an immunostimulant. Furthermore, Sarhadi et al. [58] and Zhang et al. [59] stated that plant treatment for eight weeks modulated both immunological and antioxidant qualities, which increased proinflammatory cytokines.

Antioxidant enzymes have been employed as indicators of stress factors in aquatic organisms [60]. According to Pašková et al. [61], exposure to aqueous environmental stressors might cause teratogenic alterations, oxidative stress parameters, and reproductive problems in these living organisms. Additionally, Khalil et al. [62] claimed that variations in the levels of oxidative biomarkers can serve as valuable tools for assessing pollution in aquatic systems. According to the current investigation, exposing *B. alexandrina* snails to Illicium verum significantly impacts oxidative mediators. These findings align with those reported by Ibrahim et al. [1]. Due to heightened oxidative stress and increased reactive oxygen species (ROS) production, the tissues of *B. alexandrina* snails exposed to star anise presented lower levels of antioxidants overall. This, in turn, can cause other parameters to change as well as the inhibition of antioxidant enzyme activity [63]. Additionally, oxidative stress mostly seems to be the primary factor affecting sexual maturity and the onset of delayed puberty [64]. Additionally, star anise’s effect on the mobility of membranes [65] and potential harm to DNA, proteins, and lipids [66] may be related to changes in the levels of oxidative biomarkers. Importantly, antioxidant enzymes are essential for neutralizing ROS and controlling how living organisms react to oxidative stresses [64].

The current reduction in IL-6 expression in the head foot and hepatopancreas (digestive gland) of the snails, as a result of *I. verum* treatment, suggests that anise has significant anti-inflammatory properties that are potentially beneficial in managing inflammatory conditions. These findings underscore the potential application of anise extracts as natural anti-inflammatory agents in various therapeutic applications. This potential is referred to bioactive compounds of the anise such as anethole, the primary active component in anise, along with other compounds such as estragole and anisaldehyde [5], which are known to exert anti-inflammatory effects. These compounds likely modulate inflammatory pathways by inhibiting the activation of NF-κB, which in turn reduces the transcription of proinflammatory cytokines, including IL-6 [67].

Histological examination of the head-foot and digestive glands of *B. alexandrina* treated with *I. verum* revealed significant tissue damage, including necrosis and vacuolar degeneration, which aligned with prior findings. Elhadad et al. [68] reported similar damage in snail tissues following chlorophyllin exposure, characterized by friable structures and extensive vacuolation. Similarly, Abdel-Tawab et al. [69] observed marked deterioration in digestive glands after treatment with cerium oxide nanoparticles synthesized from *Moringa oleifera*. Ibrahim et al. [70] also noted severe hepatopancreatic damage due to water-soluble chlorophyllin, whereas Dokmak et al. [71] attributed digestive gland cell breakdown in *Bulinus truncatus* to chlorophyllin-induced photosensitization.

## Conclusions

The outcome results clarified that star anise modulates the link between snail immunity and its susceptibility to overcome molluscicidal agents. Furthermore, star anise possesses highly effective active compounds that exert a novel controlling mechanism contrary to that of *B. alexandrina* snails through many mediators and gene/protein interactions. However, additional research is needed to explain the mechanism of action and its effects on other mediators related to molluscicidal/infection activities and host‒parasite interactions. The pharmaceutical and agronomy sectors are focused on creating veterinary medications that include molluscicidal and antiparasitic agents. Star anise, known for its safety and eco-friendliness, holds promise as a solution against parasites affecting aquatic organisms.

## Supplementary Information

Below is the link to the electronic supplementary material.

Supplementary file1 (CSV 5 KB)

## Data Availability

The data will be available under reasonable request.
